# The Subjective Response to Nitrous Oxide is a Potential Pharmaco-Endophenotype for Alcohol Use Disorder: A Preliminary Study with Heavy Drinkers

**DOI:** 10.1093/ijnp/pyw063

**Published:** 2016-07-11

**Authors:** Katie Walsh, Ravi K Das, Sunjeev K Kamboj

**Affiliations:** 1 Clinical Psychopharmacology Unit, Research Department of Clinical, Educational and Health Psychology, University College London, Gower Street, London (Ms Walsh and Drs Das and Kamboj)

**Keywords:** alcohol, alcohol use disorder, endophenotype, nitrous oxide, NMDA receptor

## Abstract

**Background:**

Healthy people with a family history of alcohol problems show a pattern of subjective responses to alcohol that resemble those of affected probands. Studies on ketamine suggest that up-regulation of N-methyl-D-aspartate receptors (NMDARs) underlies these effects, and point to a pharmacologically-responsive endophenotype reflecting enhanced risk for alcohol-use disorders.

**Methods:**

Subjective stimulant and sedative effects were assessed before and during nitrous oxide (N_2_O; 50%) inhalation in heavy drinkers who were otherwise healthy.

**Results:**

Participants with an ostensible family history of alcohol-use disorders (n = 23) were distinguishable from those without such familial risk (n = 37) by an enhanced stimulation-to-sedation ratio during N_2_O inhalation.

**Conclusion:**

The pattern of subjective effects of N_2_O according to familial risk is remarkably similar to that previously seen with ketamine, supporting the idea of a common, NMDAR-mediated mechanism of action. N_2_O may prove to be a safe and accessible alternative to ketamine for probing heritable NMDAR dysregulation in neuropsychiatric disorders.

## Introduction

Individuals with a positive family history (FH^+^) of alcohol use disorder (AUD) are themselves at elevated risk of developing AUD (Hesselbrock and Hesselbrock, 1992). These individuals also have a tendency to experience greater reinforcing and stimulant effects, relative to dysphoric and sedating effects, of alcohol (Morean and Corbin, 2010). Given the complex pharmacology of alcohol, delineation of the underlying neurobiology of this mixed pattern of subjective responses may seem especially challenging. However, since N-methyl-D-aspartate receptors (NMDARs) are key regulators of the reinforcing and intoxicating effects of alcohol, and are functionally up-regulated following chronic ethanol treatment in animal studies (Wang et al., 2007; see Krystal et al, 2003a), one possibility is that this stimulant effect bias reflects altered NMDARs functioning within central reward pathways (Vengeliene et al., 2008). Support for this notion is found in studies examining the effects of ketamine, a high-affinity NMDAR antagonist, in those with historic and/or familial risk of AUD. For example, recently detoxified AUD patients exhibit blunted positive and negative psychosis-like responses, as well as insensitivity to the dysphoric effects of ketamine (Krystal et al., 2003b).

A similar mixed pattern of subjective effects has been found in response to ketamine in healthy, non-dependent FH^+^ drinkers (Krystal et al., 2003a; Petrakis et al., 2004; Yoon et al., 2016). Low-affinity NMDAR antagonists also produce distinct subjective, behavioral, and neural responses in healthy individuals according to family history of AUD (Jamadar et al., 2012; Narayanan et al., 2013). These findings suggest that the alterations in NMDAR function underlying the distinct subjective responses to antagonists cannot be attributed to chronic exposure to high doses of ethanol alone (as in AUD patients; Krystal et al., 2003b).

Like ketamine, nitrous oxide (N_2_O) is a dissociative anaesthetic and primarily an antagonist at the NMDAR (Jevtović-Todorović et al., 1998). Its therapeutic potential, based on NMDAR antagonism, has only recently been explored in relation to psychiatric disorders (Nagele et al., 2015; Das et al., 2016). In the current study we draw on recent work showing an increase in the subjective stimulant-to-sedative ratio following ketamine administration in people with an inherited vulnerability to AUD (Yoon et al., 2016). We examined whether subjective responses to N_2_O—potentially also reflecting heritable NMDAR dysregulation, as above—differed in FH^+^ individuals compared to those without such histories (FH^-^). Such a pattern might suggest a common neuropsychopharmacological substrate for the subjective effects of N_2_O and ketamine. Moreover, similarities to ketamine would support the use of N_2_O as a convenient and safe pharmacodiagnostic/therapeutic agent for interrogating/treating NMDAR dysregulation in neuropsychiatric disorders. This would be an important development given that the pharmacopoeia of NMDAR-ergic agents is currently very small, its use limited (at least in the case of ketamine) by the potential for acute psychotomimetic and dysphoric effects, and the need for careful monitoring of such effects. N_2_O produces similar effects, though they are milder and reverse within minutes of terminating inhalation.

## Material and Methods

The study was approved by University College London Research Ethics Committee. All participants provided written, informed consent and all procedures were carried out in accordance with the Declaration of Helsinki code of ethics for experiments involving human subjects. The data described here are part of a larger dataset from a study on the effects of N_2_O on reconsolidation of alcohol-related memories. Participants attended three sessions separated by at least 48 hs. The data presented here is primarily from session 2, the only session during which any drug (N_2_O) was administered.

### Procedure

Prior to attendance, participants underwent a screening interview to establish eligibility. They also completed standardized alcohol-related assessments (e.g. measures of alcohol consumption and craving) and assessment of family history prior to attending session 2. After taking a breathalyzer test (Lion 500 portable Alcometer; Lion Instruments) at the beginning of session 2 (all gave a reading of 0.00), participants completed baseline subjective state measures, inhaled N_2_O for 10min, and then repeated the subjective state assessments while continuing to inhale N_2_O.

### Participants

Eligibility criteria included: an absence of current psychiatric disorder; no use of psychotropic medication; no history of drug and alcohol dependence (as assessed using the alcohol module of the Structured Clinical Interview for DSM IV); an absence of any medical contraindications to use of N_2_O; aged 18–50 years; scoring >8 on the Alcohol Use Disorders Identification Test (AUDIT; Babor et al., 2001); and regularly drinking more alcohol than the UK government recommended maximum (112 and 168g/week for women and men, respectively). As such, participants were at risk of developing AUD on the basis of current drinking patterns and AUDIT scores. This risk of transitioning from heavy to disordered alcohol use is underscored by participants’ mean age (Table 1), which is within the range of greatest relevance for such transition (King et al., 2015). Sixty individuals met criteria and attended experimental sessions. They were classified as either FH^+^ (n = 23) or FH^-^ (n = 37; see below).

**Table 1.** Demographic and Alcohol-Related Variables for Participants Without (FH^-^) and With (FH^+^) a Family History of Alcohol Problems.

	FH-	FH+
Women: n(%)	16 (43%)	7 (30%)
Age (years)	25.95 (8.14)	26.78 (9.00)
AUDIT score	13.68 (3.49)	16.35 (5.80)
Alcohol (g/week)	315.55 (149.55)	298.63 (103.53)
Tonic craving (ACQ-Now)	32.14 (8.65)	34.11 (6.81)
Picture rating (alcohol liking)	6.60 (1.07)	6.33 (1.21)
Picture rating (alcohol urge)	5.76 (1.14)	5.73 (1.26)
Depression (HADS)	2.51 (2.47)	3.04 (3.23)
Anxiety (HADS)	6.57 (3.72)	5.83 (3.34)

Except for number (proportion) of women, values are means (± standard deviations). ACQ-Now, Alcohol Craving Questionnaire-Now; AUDIT, Alcohol Use Disorders Identification Test; HADS, Hospital Anxiety and Depression Scale.

### Baseline Alcohol-Related Assessments

Baseline group differences were examined across a variety of alcohol-related measures. Alcohol consumption was assessed using the one week Timeline Followback (TLFB) procedure (Sobell and Sobell, 1992). An infographic was provided to orient participants to alcohol quantities in typical drinks (in terms of UK units), which are expressed as grams of pure alcohol in this report.

Tonic craving at baseline was assessed using the 12-item Alcohol Craving Questionnaire-Now (ACQ-Now; Singleton et al., 1994), and cue-elicited drinking urge and stimulus pleasantness, using an alcohol picture rating task. Pleasantness and urge ratings were on a 0 = “extremely unpleasant/greatly decreases” to 10 = “extremely pleasant/greatly increases” scale.

Given that exploration of heritability was a secondary aim in this study, a brief assessment of family history was deemed to be more suitable than a full familial-diagnostic interview. As such, participants were asked to indicate which, if any, relatives within the family tree of first- and second-degree maternal and paternal family members in the current and previous two generations had experienced alcohol-related problems (Mann et al., 1985). Importantly, such brief assessment methods perform nearly as well as full-scale measures of family history, with high levels of agreement between family members (Prescott et al., 2005). Participants were classified as FH^+^ if they identified either (or both) biological parent(s), or FH^-^ otherwise.

### Mood and Anxiety

The Hospital Anxiety and Depression Scale was used to gauge levels of depressed mood and anxiety (Zigmond and Snaith, 1983).

### Subjective Response to Nitrous Oxide

A 12-item visual analogue Bodily Symptoms Scale (BSS; recorded on a 0–100 scale), reflecting expected N_2_O effects, and the participant-rated items of the Clinician Administered Dissociative States Scale (CADSS; Bremner et al., 1998) were used to assess subjective states immediately before (baseline) and after 10 mins of N_2_O inhalation. Our interest related to responses to the euphoria and drowsiness items, which, following Yoon and colleagues, were expressed as a single stimulant-to-sedative ratio (Yoon et al., 2016) at baseline and during-N_2_O inhalation.

### Nitrous Oxide Administration

We administered 50% N_2_O in oxygen (Entonox, British Oxygen Company) via an Ultraflow demand valve regulator (BPR Medical Ltd) for a total of 30min. Participants remained in the department for 30min after the end of the experiment to ensure N_2_O effects had completely worn off and they were well-oriented to time and place before leaving the department.

### Statistical Analysis

Data was analyzed using SPSS version 22 (IBM) and, unless indicated, is presented as mean ± standard error of the mean throughout. Variables were examined for normality of distributions. Negatively skewed (stimulation-to-sedation ratio) data were +1 log transformed prior to a mixed factorial 2 (time as baseline; during N_2_O) x 2 (group as FH^+^ or FH^-^).

**Figure 1.**
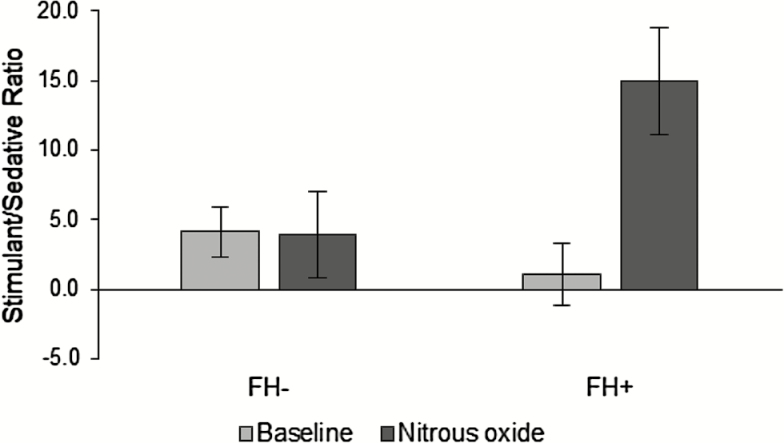
Ratio of stimulant to sedative effects of nitrous oxide (N_2_O) inhalation in participants without (FH^-^) and with (FH^+^) a family history of alcohol problems at baseline and during N_2_O inhalation. Bars represent (untransformed) estimated marginal means and associated standard errors of the means.

For ease of interpretation, raw (untransformed) values (which produced the same statistical outcome as transformed values) are shown in Figure 1. These between-/within-subject factors were also used in the analysis of CADSS data. Significant interactions were explored using Bonferroni corrected pair-wise post hoc tests. Independent sample *t*-tests were used to compare the groups’ baseline characteristics. Where the assumption of equality of variance was violated for independent sample *t*-tests, statistical correction was applied, and *t*-values and *dfs* adjusted accordingly. A small amount of missing data (~6%) is reflected in lower degrees of freedom in the reported analysis than expected for the sample size.

## Results

FH^+^ and FH^-^ groups had similar mean ages and gender composition. There were no significant differences between FH^+^ and FH^-^ groups on any alcohol- or affect-related variables (χ- or *t*-values ≤ 2; *p*-values >0.05; Table 1).

There was a significant three-way interaction between time (pre- or during N_2_O), family history (FH^+^, FH^-^), and scale [stimulation, sedation; F(1,54) = 6.98, *p* = 0.011, $\eta_{\text{p}}^{2}$ = 0.115]. Pairwise, Bonferonni corrected post hoc tests comparing pre- and during N_2_O stimulation levels showed that FH^-^ participants did not experience a significant increase in stimulation (mean change ± SEM: +7.35±6.01; *p* > 0.1), whereas FH^+^ participants did (+20.23±7.47; *p* = 0.009). Similarly, while there was a small non-significant increase in sedation among FH^-^ individuals (+5.68±5.33; *p* > 0.1), a relatively large decrease in sedation from pre- to during N_2_O (-16.36±6.23; *p* = 0.017) was found in FH^+^ participants.

To allow a more direct comparison with the pattern of effects described with ketamine (Yoon et al., 2016), the simultaneous effects of N_2_O on stimulation and sedation were also expressed and analyzed as a stimulant-to-sedative ratio. As expected, a significant Time x Group interaction [F(1,54) = 7.91, *p* < 0.01, $\eta_{\text{p}}^{2}$ = 0.128] was found on these ratio scores (Figure 1), with post hoc tests showing that an increase in stimulation-to-sedation from baseline to during N_2_O was only observed in FH^+^ participants (*p* < 0.01).

In contrast, CADSS scores showed only a main effect of Time, reflecting similar increases from baseline to during N_2_O in the FH^-^ (+15.53±2.02) and FH^+^ [+14.39±2.53; F(1,57) = 85.63, *p* < 0.01].

## Discussion

In this study we examined whether healthy individuals without alcohol dependence, but with a positive family history of alcohol problems, showed a similar pattern of subjective responses following N_2_O to those reported in other studies following ketamine administration (Krystal et al., 2003a; Petrakis et al., 2004; Yoon et al., 2016). In fact, the pattern we observed on the stimulant-to-sedative ratio measure was strikingly similar to the Group (FH^+^/FH^-^) by Time (pre-/during drug) interaction observed with ketamine (Yoon et al., 2016). It is possible that the overlapping *in vitro* neuropharmacology of N_2_O and ketamine (Jevtović-Todorović et al., 2001) underlies this similarity. More specifically, this common pattern of subjective responses in FH^+^ individuals is consistent with the notion that altered responses to dissociative-anaesthetic NMDAR antagonists are a potential pharmaco-endophenotype reflecting intergenerational transmission of dysregulated NMDAR function underlying AUDs. A full determination of the endophenotype-status of such responses requires further research, although, as with alcohol, the subjective response to N_2_O possesses at least some of the characteristics expected of an endophenotype (Morean and Corbin, 2010). Alternatively (or in addition), N_2_O response in FH ^+^ participant may reflect epigenetic influences or chronic neural dysregulation resulting from developmental adversity (arising from one or both parents’ excessive drinking). Previous research has partially addressed this concern by not including FH^+^ participants with alcohol-dependent mothers, thus at least reducing the possibility that pre-natal exposure to toxic alcohol effects contributed to differential responses in FH^+^ participants (e.g. Petrakis et al., 2004).

While we cannot rule out the possibility that the observed effects of N_2_O reflect its action on neurotransmitter systems other than NMDARs, the established receptor-level pharmacological similarities and the convergence of findings from human psychopharmacological studies across various NMDAR antagonists makes an NMDAR-mediated account of the current findings the most parsimonious. If our findings do reflect the action of N_2_O on a heritable dysregulated NMDAR system, they suggest that research participants are reliably able to identify this dysregulation through its ultimate expression: namely, in the behavior of their relatives. In addition, the results potentially indicate that expression of NMDAR dysfunction in FH^+^ individuals is associated with a lower threshold of problem severity (among relatives) than has been implied by previous studies with ketamine, in which FH^+^ participants were those with alcohol-dependent family members (Petrakis et al., 2004; Yoon et al., 2016).

Our findings have potentially important clinical implications. For example, an appraisal of family history of alcohol problems may be important when determining the suitability of N_2_O (or other NMDAR antagonists) as a treatment for depression (Phelps et al., 2009; Nagele et al., 2015), post-traumatic stress disorder (Das et al., 2016), and addictions (David et al., 2006). Given the recent surge of interest in NMDAR antagonists as novel pharmacotherapeutics for major depressive disorder (Coyle and Laws, 2015; Nagele et al., 2015), an improved understanding of predictors of NMDAR antagonist responses will be key for understanding variability in treatment and may facilitate the development of personalized interventions for depression. Despite these possibilities, it is important to extend our preliminary findings. For example, a future study should use standardized assessments of subjective drug effects, and assess the similarity of N_2_O’s effects to those of alcohol (e.g. using the biphasic alcohol effects scale; Yoon et al, 2016).

In sum, the subjective response to N_2_O may be an efficient and convenient pharmacological probe that signals NMDAR dysfunction and in turn, increased risk of neuropsychiatric disorders or probable treatment response. While evidence mounts that ketamine is a valuable and potent probe drug and pharmacotherapeutic agent, there continues to be a need for effective, easily-administered, and rapidly-reversing NMDAR antagonists for use in the prognostics and treatment of neuropsychiatric disorders. N_2_O appears to have these properties.

## Statement of Interest

None.
